# *Talaromyces marneffei* infection in a lung cancer patient: a rare case report

**DOI:** 10.1186/s12879-019-3968-5

**Published:** 2019-04-24

**Authors:** Fanhai Lin, Ye Qiu, Wen Zeng, Yi Liang, Jianquan Zhang

**Affiliations:** 1 Department of Respiratory Medicine, The First Affiliated Hospital of Guangxi Medical University, Nanning, 530021 Guangxi China; 2 Department of Comprehensive Internal Medicine, The Affiliated Tumor Hospital of Guangxi Medical University, Nanning, Guangxi China

**Keywords:** *Talaromyces marneffei*, HIV-negative, Primary pulmonary lymphoepithelioma-like carcinoma

## Abstract

**Background:**

*Talaromyces marneffei* is an invasive, and thermal dimorphic pathogenic fungus, whose infection is life threatening in human*.* Although immunocompromised patients, such as patients with human immunodeficiency virus infection and recipients of organ transplant, are susceptible hosts, infections have been recently reported in people with normal immune function. Patients with cancer may also be susceptible hosts but no case of *T. marneffei* infection has been reported in patients with lung cancer. In this case, we describe *T. marneffei* infection coexisting with primary pulmonary lymphoepithelioma-like carcinoma (LELC) in an HIV-negative patient.

**Case presentation:**

A 50-year-old, previously healthy female presented with a 1-month history of cough and fever. CT scans showed a mass in the left lower lung, left pleural thickening, pleural effusion, and multiple swollen lymph nodes throughout the body. Based on the pathology of the left lung lesion, she was diagnosed with left primary pulmonary LELC complicated with *T. marneffei*. She received both anti-tumor and anti-fungal treatments. A subsequent CT re-examination demonstrated that the mass was absorbed remarkably after treatment. Follow up showed no tumor progression and no relapse of *T. marneffei* infection.

**Conclusion:**

This case suggested that clinicians should pay more attention to the potential hosts of *T. marneffei* infection, especially those with lung cancer. Early diagnosis and treatment can improve the prognosis of *T. marneffei* infection coexisting with lung cancer.

## Background

*Talaromyces marneffei*, causing talaromycosis marneffei, is a highly opportunistic fungal pathogen, most commonly reported in patients with HIV infection, and recently reported in individuals with normal immune function [1]. Patients with cancer may also be susceptible hosts. The infection caused by this pathogen in the bronchus and lungs appear similar to lung cancer in imaging and bronchoscopy. We observed that *T. marneffei* is easily disseminated during tumor treatment without timely diagnosis. Herein, we report *T. marneffei* infection coexisting with primary pulmonary lymphoepithelioma-like carcinoma (LELC), a rare subtype of non-small cell lung cancer, in a female patient. She was successfully treated with antimicrobial therapy and antineoplastic drugs. To the best of our knowledge, this is the first such reported case.

## Case presentation

A 50-year-old, previously healthy female was presented to our institute to further investigate sputum-coughing, fever, and pulmonary shadow on October 25, 2016. The patient used to be exposed to a work-related humid environment but had no smoking history. The female presented a 1-month history of coughing, fever (highest temperature 37.4 °C), and weight loss (4 kg). She was admitted to a local hospital, and computed tomography (CT) of the chest revealed a mass in the left lung. Lung cancer and obstructive pneumonia were confirmed by clinical diagnosis. However, her condition did not improve after antibacterial treatment.

**Fig. 1:**
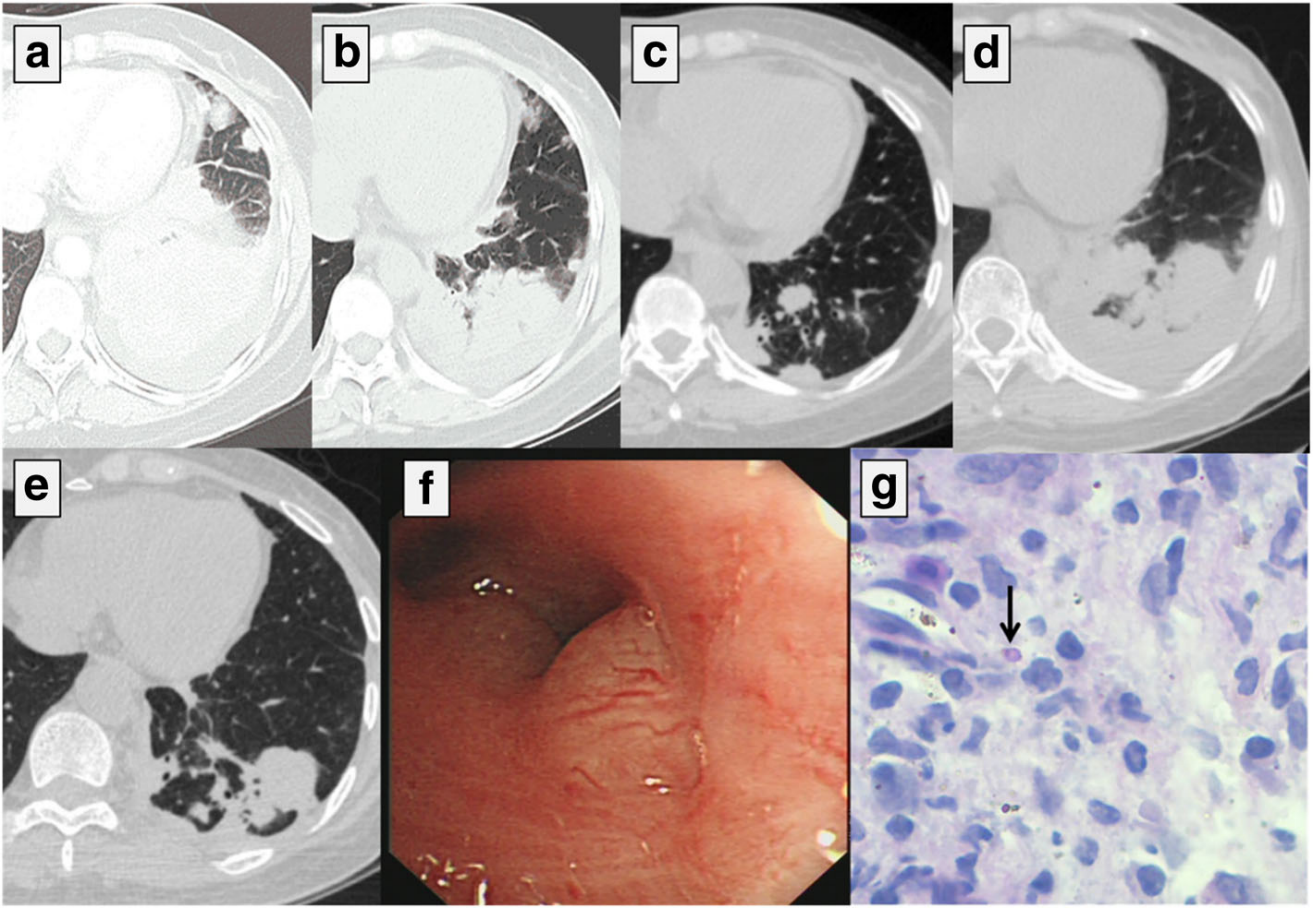
Chest CT (**a**-**e**) **a**) a mass and pleural effusion in the left lower lung on October 28, 2016. **b**) The mass in the left lower lobe was obviously smaller than that **a**) on November 2, 2016. **c**) The size of the nodule in the left lung tended to be stable with little change on April 26, 2017 (seven and a half months after taking apatinib). **d**) The nodule in the left lung progressed nine and a half months after taking apatinib. **e**) The lesions significantly improved on September 4, 2018. **f**) Bronchial stenosis with nodular projections. **g**) Yeast-like cell could be identified in the endobronchial nodule (PAS original magnification × 1000)

On examination, an enlarged supraclavicular lymph node was observed on the right. The lower left lung revealed low breath sounds. The serum carbohydrate antigen 125 (CA125), CA153, and CA19–9 levels were 94.5 (reference 0–30.2 U/mL), 37.2 (reference 0–32.4 U/mL), and 49.9 U/mL (reference 0–37.0 U/mL), respectively. The blood was HIV negative. The CD4+ and CD8+ T-lymphocyte counts were 744 and 576 cells/μL, respectively. The plasma galactomannan test result was positive. The level of C-reactive protein was 7.68 mg/L. However, the serum cryptococcal antigen agglutination test, acid-fast bacillus test of sputum, bronchoalveolar lavage fluid smear, and blood and sputum cultures for fungus and bacteria were negative. The serum level of immunoglobulin (Ig)G, IgA, and IgM was normal. Other routine laboratory tests were normal, including the complete blood count, blood sugar, aspartate aminotransferase, alanine aminotransferase, creatinine, antinuclear antibodies, extractable nuclear antigen antibodies, anti-neutrophil cytoplasmic antibodies, anti-ds-DNA antibodies, and cardiolipin-antibodies. The mass identified by CT in the left lower lung (Fig. 1a), was accompanied by left pleural thickening, pleural effusion, and multiple swollen lymph nodes throughout the body, while no abnormal brain CT, liver B-ultrasonic, and emission CT images were observed. Bronchoscopic examinations showed evident nodular projections in the bronchus orifices of the left lower lobes (Fig. 1f). Lymphoepithelioma-like carcinoma and chronic granulomatous inflammation were found by histological examination of the endobronchial nodule. Immunohistochemistry revealed that the cytokeratin (CK) CK5/6, P40, and Epstein–Barr virus-encoded RNA were positive, whereas TTF-1, p63, CK7, CK56, syn, and epidermal growth factor receptor (EGFR) were negative. Pharyngorhinoscopy and pathology of the nasal mucosa showed no tumor. Several yeast-like organisms with red cell wall and clear PAS-negative cell content were identified in the biopsy tissue (Fig. 1g). We concluded a definite diagnosis of primary pulmonary lymphoepithelioma-like carcinoma with *T. marneffei* infection.

Her condition improved after a week of treatment with voriconazole (200 mg IV) at every 12 h. Docetaxel (120 mg) and carboplatin (600 mg) were prescribed as the first cycle of chemotherapy. Pulmonary shadow was significantly improved (Fig. 1b) and the size of mediastinal lymph nodes reduced. The patient was prescribed oral itraconazole therapy and discharged. However, myelosuppression occurred during the fifth cycle of chemotherapy and fungal disease subsequently relapsed. She was unwilling to undergo further chemotherapy because of the poor tolerance and attempted to take apatinib orally. The pulmonary lesions remained stable until nine and a half months (Fig. 1c, d). Percutaneous lung biopsy revealed lymphoepithelioma-like carcinoma but no *T. marneffei*, which suggested tumor relapse and talaromycosis marneffei cure. Itraconazole treatment was withdrawn and a single dose of docetaxel (120 mg) was prescribed.

The patient is still being followed up (Fig. 1e).

## Discussion and conclusions

*T. marneffei* is an opportunistic pathogen common among HIV-infected patients. However, an increasing number of *T. marneffei* infection in non-HIV-infected patients has recently been reported, especially in those with hematological malignancies, autoimmune diseases, organ transplantation, and diabetes mellitus [1], but not in those with lung cancer. This is the first case report describing *T. marneffei* infection in a non-HIV-infected patient with existing pulmonary LELC.

*T. marneffei* can invade multiple organs including the lung, skin, liver, bone, trachea and bronchus, and brain, like lung cancer. Patients may present respiratory symptoms including fever, cough, and expectoration involving the trachea and bronchus. The lesion in the chest imaging displays as single or multiple lobar consolidation, mass, cavity, interstitial exudation, pleural effusion, and pericardial effusion, commonly accompanied by hilum and mediastinal lymph node enlargement [1]. Tracheoscopy shows tracheal and/or bronchial nodules or masses, thickened mucosa, and uneven, narrow lumen [2, 3]. Indeed, the similarities in terms of clinical manifestations, chest imaging and bronchoscopy findings between talaromycosis marneffei and lung cancer are remarkable. The diagnosis of *T. marneffei* infection mainly relies on tissue culture and pathological examination. *T. marneffei* is thermally dimorphic, growing as a mycelium at 25 °C and as yeast-like cells at 37 °C on Sabouraud dextrose agar, exhibiting the production of soluble red pigment that diffuses into the medium. Furthermore, *T. marneffei* yeast-like cells, which are 3–8 μm in diameter, could be observed by periodic acid-Schiff stain, revealing a transverse septum or sausage-shaped form that is the characteristic morphology [4]. In this case, round to oval yeast-like cells with a transverse septum were observed in the left lung lesion. Ultimately, the patient was diagnosed with left primary pulmonary LELC complicated with talaromycosis marneffei. However, several manifestations primarily misled us toward lung cancer, ignoring *T. marneffei* infection. Further, it was difficult to obtain evidence of the infection because of limited viable *T. marneffei* in HIV-negative biopsy tissues and the low, positive rate of culture of *T. marneffei*. Therefore, pathogens should be carefully searched for in pathological examination. Our patient presented fever and bronchial pathological evidence, and displayed chronic granulomatous inflammation. Some infections such as those of *Mycobacterium tuberculosis*, non-*Mycobacterium tuberculosis*, *Aspergillus*, and *T. marneffei* need to be considered and specific staining such as acid-fast stain, PAS, or others should be conducted for differential diagnosis.

Itraconazole, amphotericin B and voriconazole are effective in treating talaromycosis marneffei [5, 6]. Pulmonary LELC is sensitive to paclitaxel- or docetaxel-based regimens [7] and apatinib can be used as a third-line treatment for EGFR wild-type advanced non-small cell lung cancer [8]. Our patient received voriconazole and subsequently docetaxel + carboplatin. During the first cycle of chemotherapy, exactly 20 days after beginning the treatment with voriconazole, the mass in the left lower lung was significantly reduced, which may be a result of the antifungal therapy. However, talaromycosis marneffei recurrence was caused by bone marrow suppression in the course of chemotherapy. Missed diagnosis of *T. marneffei* in lung cancer can be fatal as the infection would spread owing to decreased white blood cell levels after chemotherapy. Thus, apatinib was approved for lung cancer with no white blood cell level reduction, which avoids inducing talaromycosis marneffei relapse.

In summary, diagnosis of lung cancer with easy access to pathological evidence is relatively simple, whereas diagnosis of talaromycosis marneffei is challenging. Missed diagnosis of talaromycosis marneffei may result in the fatal spread of the fungus during anti-tumor treatment. Therefore, the timely diagnosis and treatment of talaromycosis marneffei is critical, and attention should be paid by clinicians and pathologists.

## Abbreviations

CA: Carbohydrate antigen
CK: Cytokeratin
CT: Computed tomography
EGFR: Epidermal growth factor receptor
Ig: Immunoglobulin
LELC: Lymphoepithelioma-like carcinoma
